# Insurance barriers and inequalities in health care access: evidence from dual practice

**DOI:** 10.1186/s13561-024-00500-y

**Published:** 2024-03-21

**Authors:** Eva Goetjes, Katharina E. Blankart

**Affiliations:** 1 CINCH Health Economics Research Center, University of Duisburg-Essen, Berliner Platz 6-8, 45127 Essen, Germany; 2 Leibniz Science Campus Ruhr, Essen, Germany; 3 School of Health Professions, Institute of Health Economics and Policy, Bern University of Applied Sciences, Bern, Switzerland

**Keywords:** Physician treatment style, Health insurance, Practice composition, Equity of care, I10 Health General, I11 Analysis of Health Care Markets, I13 Health Insurance, Public and Private, I18 Government Policy, Regulation, Public Health

## Abstract

**Background:**

We investigate access disparities in pharmaceutical care among German patients with type 2 diabetes, focusing on differences between public and private health insurance schemes. The primary objectives include investigating whether patients with private health insurance experience enhanced access to antidiabetic care and analyzing whether the treatment received by public and private patients is influenced by the practice composition, particularly the proportion of private patients.

**Methods:**

We estimate fixed effect regression models, to isolate the effect of insurance schemes on treatment choices. We utilize data from a prescriber panel comprising 681 physicians collectively serving 68,362 patients undergoing antidiabetic treatments.

**Results:**

The analysis reveals a significant effect of the patient's insurance status on antidiabetic care access. Patients covered by private insurance show a 10-percentage-point higher likelihood of receiving less complex treatments compared to those with public insurance. Furthermore, the composition of physicians' practices plays a crucial role in determining the likelihood of patients receiving less complex treatments. Notably, the most pronounced disparities in access are observed in practices mirroring the regional average composition.

**Conclusions:**

Our findings underscore strategic physician navigation across diverse health insurance schemes in ambulatory care settings, impacting patient access to innovative treatments.

**Supplementary Information:**

The online version contains supplementary material available at 10.1186/s13561-024-00500-y.

## Introduction

Use of the latest technology is linked to productivity growth and improvement in patient health. At the stage when health technologies are established, persistent adoption of innovation is driven more by physicians and providers than by knowledge diffusion [1, 2]. Such unwarranted variation in physician treatment styles may be driven by differences in health insurance schemes and may vary by composition of patients per health insurance scheme in a practice. This can result in inequalities in access to cost-effective innovation and can have severe consequences on patient health, and efficiency of health care systems.

We analyze the impact of health insurance schemes on choices that determine access to novel treatments with less complex medication regimen when physicians work in dual practice. Treatment choices can have important consequences for patient health and efficiency of health care delivery. In health care settings with mutually exclusive insurance schemes, where physicians can freely choose between treatments that may vary by quality and cost-effectiveness, and patients can freely choose physicians, policymakers would like to know whether mechanisms are needed to regulate treatment styles if access to more novel but effective treatments is not equal per insurance scheme.

Miraldo et al. [1] suggest that to understand the role of the physician in technology adoption, any investigation of differences in access beyond the physician’s office door needs to account for differences in treatment style of physicians and patient-level factors. Access to prescription medicines is determined by the physician’s decision and can only be obtained from seeing a physician who diagnoses medical conditions and subsequently decides on treatment. Our approach complements previous studies that have investigated the effects of insurance schemes on access by waiting times to see a provider by studying the effects on obtaining prescription medicines by health insurance status and the within practice variation in prescriptions [3–5].

Delaying access to care of effective prescription medicine because of a patient’s health insurance scheme may lead to the loss of potential health benefits, ultimately lower life expectancy as well as preventable costs arising from non-adherence [6, 7]. This is of particular interest for chronic conditions with a progressive nature like type 2 diabetes (T2D), as most patients at some point need more than one active ingredient to ensure long-term glycemic control. Patients have a significantly higher adherence to medicine regimen with few tablets (i.e. monotherapy) [8, 9], and that patients changing from a single- to a two-pill regimen leads to higher adherence (71% vs. 87%) [10]. Our study expands the literature by capturing unwarranted treatment variation when choosing between technologies of different complexity within the same practice.

This study focuses on physicians working in dual practice, offering services to two types of mutually exclusive health insurance schemes in Germany, public and private. Important to note is, that there are no direct incentives for physicians to differ their prescribing between patients with public and private health insurance. We analyze physician prescription decisions between treatments for patients with T2D receiving oral antidiabetic therapy. First, we study whether patients with private health insurance are more likely to receive a less complex single-pill treatment compared to a two-pill treatment. Second, we analyze whether the treatment of publicly and private patients with T2D depends on practice composition, hence the share of private patients in the practice.

We find evidence of unequal access to prescription medicines depending on the patient’s insurance scheme. Private patients are about 10 percentage points more likely to receive the less complex single-pill treatment compared to a two-pill treatment. We document significant inequalities in treatment choices by practice composition. The beneficial effect of the private insurance status on access to the single-pill is, with about 12 percentage points, larger in a practice with a high relative share of private patients. The inequalities are highest when the share of private patients is most similar to that of the regional average of the respective federal state.

## Conceptional and institutional background

### Private and public health insurance in Germany

We compare differences in access between the two mutually exclusive health insurance schemes of non-profit public health insurances and private health insurances [11]. In Germany, health insurance is compulsory for all citizens. 87.7% of the population are insured in a public insurance and about 10.5% in a private insurance [12]. The eligibility criteria and financing of the insurance schemes differ. Employees whose income does not exceed a compulsory insurance threshold (€5,212.50 in 2020) obtain insurance by one of the 103 sickness funds of a public insurance [13, 14]. If the monthly income exceeds the threshold, citizens can voluntarily opt into one of the plans provided by 44 companies that offer a private insurance [15]. Civil servants and self-employed citizens are insured with a private insurance, even below the compulsory income threshold.

With respect to financing, public insurance is based on a solidarity-based health system. Contributions are calculated based on income, including family members of insured individuals and the retired having access to the same benefits [11]. This contrasts with private insurance where premiums are determined according to health status, age, and individual risk assessment when entering the system [4, 13, 16]. Switching from public insurance to private insurance is possible given eligibility criteria [17].

The provision of ambulatory care services in both health insurance schemes is characterized by fee-for-service reimbursement. Physicians are largely autonomous when making prescription decisions compared to health systems with managed care or a national health service that often restrict prescription choice [18]. Regarding prescription medicines, the scope covered by public and private insurance is almost identical. All active ingredients that received marketing authorization can generally be prescribed in public insurance unless listed on a rather narrow negative list [19]. What differs are the incentives to prescribe novel treatments.

### Access to technology per insurance scheme

We study two channels how health insurance schemes influence treatment decisions in dual practice settings: financial incentives and practice composition in dual practice. Both channels are indirect, as upfront there are no restrictions to prescribe medicines at the level of a single prescription in both, the public and private health insurance scheme as long as the medicines are not listed on a negative list. Dual practice refers to a physician treating patients of different health insurance schemes simultaneously in the same practice [20]. In Germany, about 97% of the ambulatory physicians work in dual practice [21] and working in this setting has been associated with prioritization of patients due to financial interests, differential waiting times, patient selection according to profitability, over- or under provision of healthcare ultimately impacting the health outcomes [5, 20, 22–25].

### Financial incentives to unequal access per insurance scheme

We examine financial incentives as a channel to choose a more novel and potentially costlier treatment in one group of patients per health insurance scheme, but not the other. Such financial incentives originate from differing physician compensation depending on patient types visiting the practice and the management of prescription medicine spending by insurance scheme.

The major difference between the public and private insurance schemes is that public insurance monitor prescription expenditures using cost control measures which may lead to different but indirect financial incentives. These measures include a quarterly budget and prescription quotas based on the physicians’ specialization and number of patients in the previous year. The quarterly budget and prescription quotas are monitored at the practice level [21, 26, 27]. When the physician reaches the quarterly budget, additional services and prescriptions are only reimbursed partially [21]. If physicians do not comply with the control measures, they face the risk of a recourse claim of expenditures beyond the budget. Prescribing expensive medicines, will lead to a physician overspending her budget quicker. To stay in the budget, thus to optimize her compensation the physician is incentivized to limit ambulatory services provided and the prescription of expensive medicines if cheaper alternatives are available. For private patients, no such cost-control measures exist. The private insurance is based on a fee-for-service basis and the patients pay their services up-front by entering into a contract with the physician and paying physicians directly after receiving an invoice. The set of reimbursable services is listed with a uniform baseline price across different private plans though can be multiplied with leverage factors depending on the complexity of treatment and time spent with the patient. Ultimately, there is no cap on service or prescription volume in private insurance and comparing similar services across these two reimbursement schemes, charges of physicians were, on average 2.28 times higher for privately insured compared to publically insured patients [21, 28]. The difference in reimbursement rate is relevant in the compensation of an ambulatory care physician of which about 22% stems from the about 10% of privately insured or self-paying patients in the practice [29].

Given the financial incentive and institutional differences regarding cost-control measures that apply to public but not to private insurance in Germany, we will empirically test the hypothesis whether patients with private insurance are more likely to obtain access to more novel treatments compared to patients with public insurance. If we do not find such differences, it may be that physicians put value on treating patients according to the same criteria which may be an intrinsic value to physicians [30]. Such notions of fairness would constrain profit-maximizing choices of physicians so that we would not find differences in technology use across health insurance schemes. If the opposite is the case, we would conclude that the differences in financial incentives and cost-control measures of the German public insurance scheme is a factor that leads to different decision-making structures of physicians [31].

### Practice composition and treatment choices

Practice composition captures the proportion of patients of different health insurance schemes treated in a practice which indicates how much a physician is exposed to a certain regulatory framework. Practice composition may also reveal how much a physician may need to customize a treatment for a given patient population. In settings with mutually exclusive insurance schemes, physicians typically see patients from a variety of arrangements by public or private insurance schemes that vary in payments and management of patients. We will test whether a higher share of private patients in the practice increases the publicly insured patient's likelihood to receive the less complex treatment in a setting where the vast majority of patients is insured in the public insurance scheme.

Previous literature has almost exclusively analyzed the effect of practice composition on practice intensity and efficiency in the context of traditional fee-for-service settings compared to managed care in the United States [32–34]. The setting we study does not differ by payment type, but management and monitoring of prescription medicine spending at physician level, most importantly delivery arrangements when there is no direct financial gain, loss or additional cost from prescribing a medicine.

For the German setting, we consider practice composition by the share of how much a physician relies on either insurance scheme as indicator of how much costs differentiated into fixed and variable costs is allocated to the patients by insurance scheme. Physicians tend to shift their resources onto private patients to compensate for lower expected reimbursement by a larger share of public patients [35, 36]. We consider physicians trying to limit variable costs to be less likely to adopt a new technology and that practice composition is indicative of the strength of the physician’s profit maximizing motive.

An important aspect regarding practice composition concerns how the practice composition differs from the practice composition of physicians practicing in the same region. That way we can analyze whether physicians stick to a norm following behavior in which physicians optimize their decisions at the aggregate level for a representative patient instead of taking decisions individually per patient [37]. If physicians stick to a certain norm, this means that physicians choose treatments for the median or mean patient in their practice and not customize treatments. Such a strategy may be sensible to reduce the cost of customization that have been related to behavioral factors and heuristics [31]. If the share of privately insured is relatively higher compared to regional average, physicians may adapt their treatment choices such that patients of public insurance will also be more likely receive that treatment that represents the standard for the privately insured. We assume that physicians in practices with a higher share of privately insured than the regional average to have a different practice style than physicians with a relatively low share of privately insured and that due to costs of customization, the treatment of publicly insured will resemble the treatment of the privately insured such that more novel therapies are prescribed for the publicly insured.

### Oral antidiabetic care of T2D patients

We consider patients suffering from T2D which are treated with a novel combination therapy of oral antidiabetics (i.e. dual therapy). In Germany, around 7 million people were diagnosed with diabetes in 2015, with an estimated 140,000 annual deaths in 2018 [38–40]. T2D prevalence is continuously rising and forecasts predict an increase by 54‒77% by 2040 [40]. The global healthcare spending for the treatment of diabetes was estimated at 673 billion US Dollar in 2015 [41], indicating a high financial burden. In Germany, expenditures for oral antidiabetics were growing by 7‒11% annually with a total value of 1.1 billion Euros in 2018 [42]. Moreover, diabetes is a significant risk factor for several cardiovascular diseases [43].

We concentrate on oral antidiabetic treatments comprising approved active ingredients for managing hyperglycemia, aimed at adjusting a patient's blood glucos level. The standard therapy after diagnosis involves a patient’s lifestyle change (e.g. weight loss, smoking cessation). If such measures fail to achieve the desired outcomes, and if there is no contraindication, oral antidiabetic therapy is initiated [44]. There are eight prescription medicine classes available: biguanides (thereof most importantly metformin), sulfonylureas, glinides, glitazones, glucagon-like peptide-1 receptor agonists (GLP-1-agonists), sodium-glucose cotransporter 2 inhibitors (SGLT2-inhibitors), dipeptidyl peptidase IV inhibitors (DPP-IV-inhibitors), and alpha-glucosidase inhibitors [45]. The different ingredients aim to alter blood glucose levels, but work through different mechanisms [46]. The variety of active ingredients is meaningful both for patients with comorbidities, or intolerances towards certain active ingredients, as well as in dual therapy, as it shows a potentially complementary and additive effect of the different mechanisms [47]. According to clinical treatment guidelines [44], metformin is the ingredient of first choice. In patients where a monotherapy using solely metformin is not achieving desired treatment outcomes, as well as with the progression of the disease, metformin can be combined with one or multiple active ingredients of eight oral antidiabetics classes.

Based on the clinical evidence proving the effectiveness in T2D therapy, we assume dual therapy to be more effective than monotherapy [48, 49]. Within dual therapy, we assume that a single-pill is superior to a two-pill treatment [48, 49]. A commonly used reference point for evaluating the effectiveness of medicines in T2D therapy is the change in blood glycated hemoglobin levels (HBA1c). A randomized clinical trial found significantly lower HBA1c levels in patients receiving dual therapy of metformin and DPP-IV-inhibitors compared to patients with metformin monotherapy [47]. A recent randomized clinical trial by Rosenstock et al. [50] indicates significantly lower HBA1c levels in patients receiving dual therapy of metformin and GLP-1-agonists compared to patients receiving dual therapy of metformin and DPP-IV-inhibitors.

## Methods

### Data sources and study sample

We combine data from two sources. To capture physician prescribing decisions, we rely on the CEGEDIM MEDIMED prescriber panel. The dataset contains prescription data of 3,026 office-based physicians in Germany over the years 2011 to 2014. It is a representative sample covering about two percent of all practicing physicians registered in Germany. The panel includes details about the prescription, selected patient characteristics including insurance scheme, and selected physician characteristics. Compared to administrative claims data, the panel allows comparing a physician’s treatment behavior of patients from both public and private insurance in the same practice. To classify products by ingredient, we rely on the EphMRA/PBIRG Anatomical Classification for information on active ingredients [45].

### Study Sample

We narrow our sample to patients who use ‘Drugs used in Diabetes’ (A10H – A10S according to EphMRA) to be able to assume similar prescription behavior [45]. We focus our analysis on oral antidiabetics that account for about 2.69% of prescriptions in ambulatory care and belong to the more frequently prescribed medicines. We exclude insulin products, both animal and human, and medical devices used for antidiabetic care.

We included patients of age 18 and older who have received an oral antidiabetic treatment based on dual therapy (i.e. metformin and DPP-IV-inhibitors or glitazones or sulfonylurea), excluding patients who receiveonly one active ingredient. No patient changed insurance scheme during the observation period. With respect to physicians, we included general practitioners and internal medicine specialists working in dual practice (i.e. treating both, publicly and privately insured patients) and treating a minimum of 60 patients with oral antidiabetics across the whole observation period. Our final analysis sample includes 979,949 prescriptions for 68,362 patients prescribed by 681 physicians.

We calculate the share of private patients in a practice and the average share of private patients in a practice at regional level the practice is located in. The regional level is divided in 17 physician association (*“Kassenärztliche Vereinigungen”*) regions that are represented by the 16 federal states and the state of North-Rhine Westphalia split in two regions. We calculate the relative share of private patients within one practice compared to the average share by physician association region. The relative share is the difference between the regional average and the absolute proportion in a practice, with positive values indicating that a practice has more private patients than the regional average. We classified patients’ comorbidities using a prescription based risk score based on 46 comorbidity classes classified in Pratt et al. [51], corresponding with the codes of the WHO’s Anatomical Therapeutic Chemical (ATC) classification.^1^

### Summary statistics

Table 1 shows summary statistics of the study sample stratified by insurance scheme and treatment choice. We focus on initial prescription decisions of T2D dual therapy. Compared to publicly insured, privately insured have a ten percentage point higher share of receiving the single-pill. Patient characteristics suggest that T2D patients do not differ strongly by insurance scheme. Private patients are more often treated by specialist physicians (42% vs. 33%), this could be due to regional variation of both, share of private patients as well as specialists. Urban regions have larger proportions of private patients and specialists than more rural regions or regions in the former East-German area [21]. (Table 1)

**Table 1:** Descriptive Statistics of patients using oral antidiabetics, total and stratified by insurance scheme

Variable	Definition	Total		Public		Private	
		mean	sd	mean	sd	mean	sd
Single-pill	1 if single-pill	0.58	0.49	0.57	0.49	0.70	0.46
*Insurance*							
Public	1 if Public	0.94	0.24	1.00	0.00	0.00	0.00
Private	1 if Private	0.06	0.24	0.00	0.00	1.00	0.00
*Patient characteristics*							
Pat Age	Age patient	66.20	11.74	66.25	11.81	65.34	10.59
Risk Score	Risk score patient	4.87	2.38	4.89	2.39	4.50	2.27
Patient Sex							
Female Pat	1 if female	0.44	0.50	0.46	0.50	0.23	0.42
Male Pat	1 if male	0.56	0.50	0.54	0.50	0.77	0.42
*Physician characteristics*							
Doc Age	Age physician	57.99	7.05	58.01	7.04	57.66	7.17
Physician Sex							
Female Doc	1 if female	0.24	0.42	0.24	0.43	0.15	0.35
Male Doc	1 if male	0.76	0.42	0.76	0.43	0.85	0.35
Specialist	1 if specialist	0.34	0.47	0.33	0.47	0.42	0.49
PHI Share							
< 5%	less than 5%	0.55	0.50				
5–10%	between 5 and 10%	0.28	0.45				
10–15%	between 10 and 15%	0.11	0.32				
> 15%	more than 15%	0.06	0.23				
Relative share	relative PHI share	-0.22	4.37				
*N (patients)* *N (physicians)*		68,362 681		64,195 681		4,167 628	

Data source: CEGEDIM MEDIMED prescription data 2011–2014

*Abbreviation: PHI*, Private Health Insurance

**Fig. 1:**
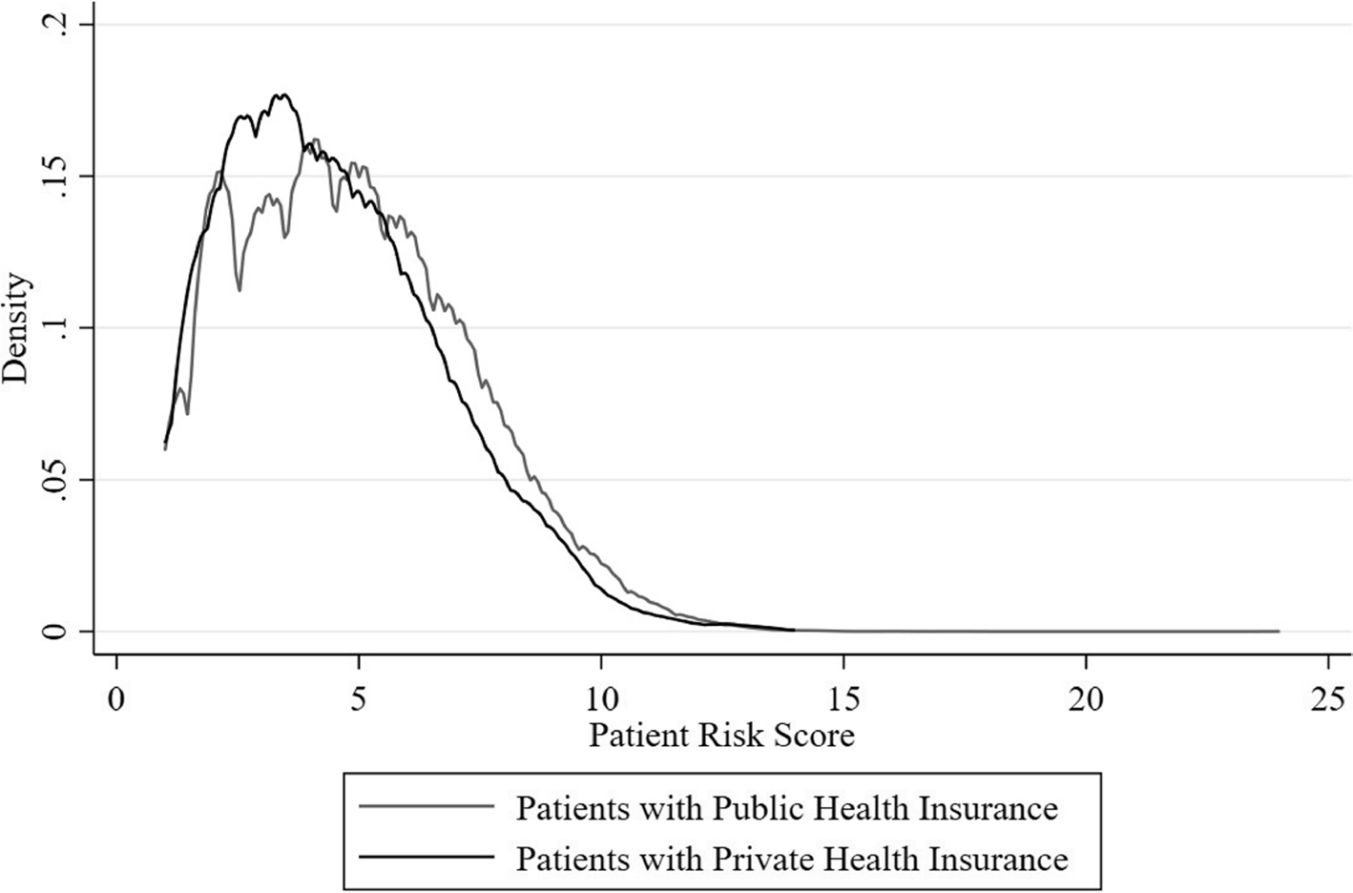
Mean patient comorbidity risk score by health insurance scheme

Figure 1 shows the density of the mean risk score of patients included in our sample stratified by health insurance scheme. There is a large overlap in comorbidity profiles across the distribution of risk scores with a larger proportion of publicly insured that have higher risks scores.

**Fig. 2:**
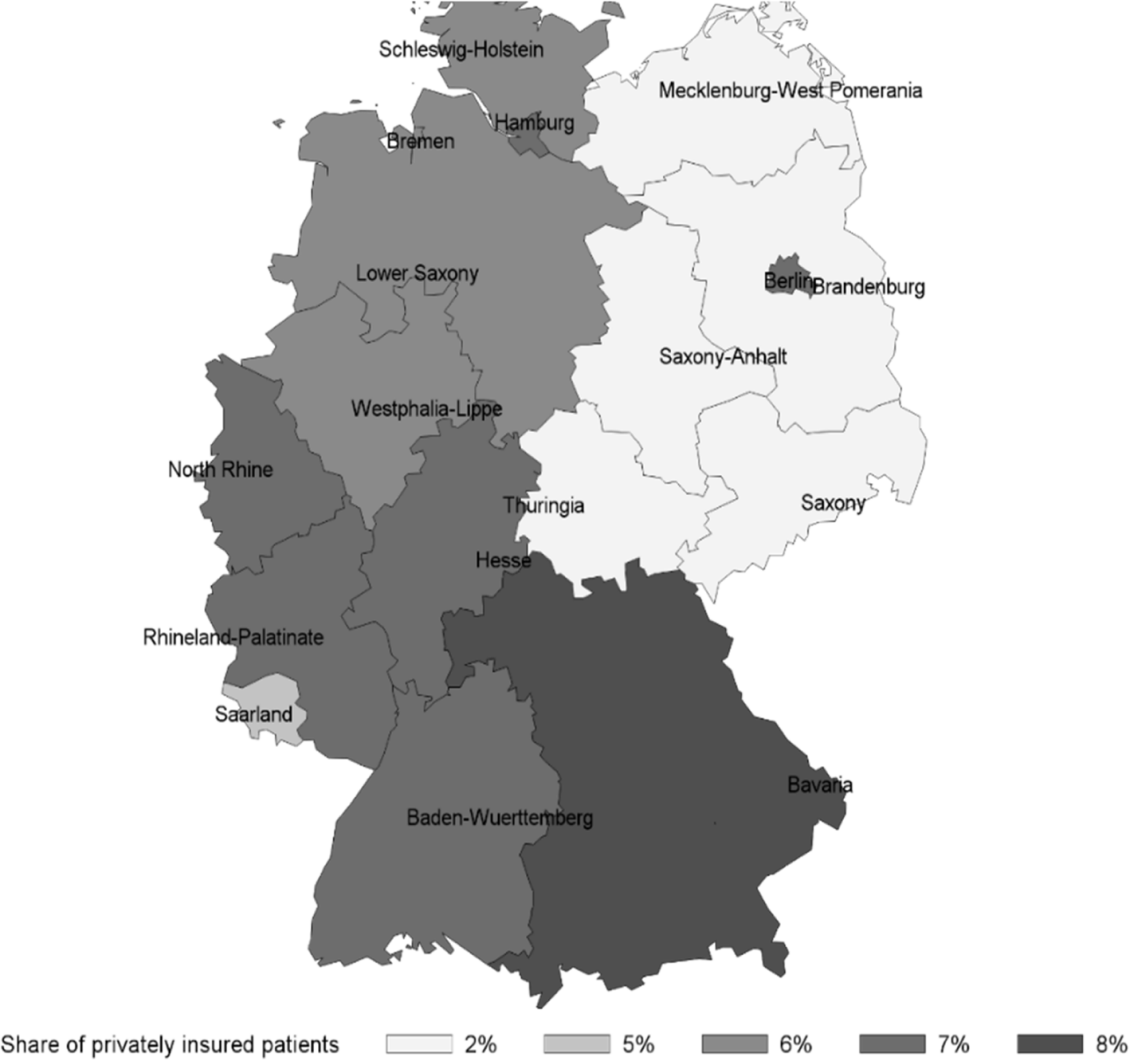
Share of private patients receiving oral antidiabetic treatment in dual practice, 2011–2014. Data source: CEGEDIM MEDIMED prescription data 2011–2014

Figure 2 shows the regional variation of the share of private patients receiving oral antidiabetics. This share varies substantially between 2 and 8% and is lower in Eastern states of Germany compared to Western parts. The North-Eastern parts correspond with the former territory of the German Democratic Republic where the share of privately insured is historically lower. The regional variation suggests that the profit maximization motive from treating private patients in dual practices could vary across regions [22]. As providing services to private patients is generally more profitable to physicians [28, 29], we assume that profit maximization is easier to achieve in areas where the share of private patients is higher.

**Fig. 3:**
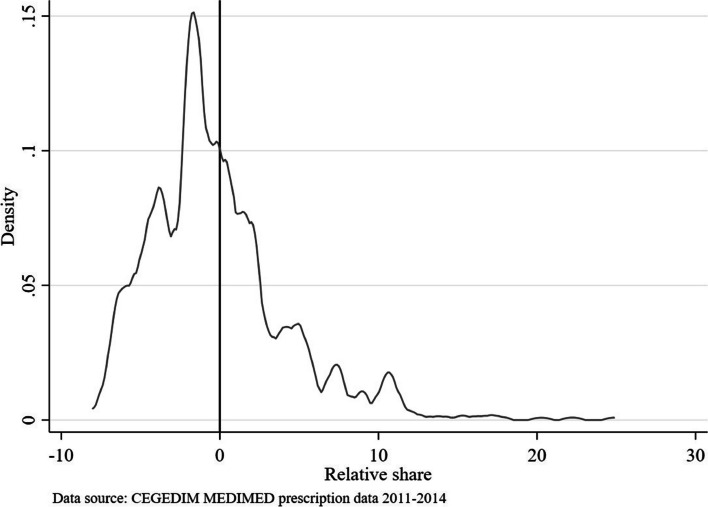
Regional relative share of private patients in practice relative to regional average. Note: The relative share shows the difference in the share of private patients in the practices relative to the regional average at the level of a physician practice

Figure 3 shows the density of the difference in the share of private patients in the practices relative to the regional average. Positive values indicate that a practice has more private patients than the regional average. The distribution of the regional relative share suggests that the majority of practices have less private patients than regional average, that means values below zero. The relative share of private patients shows an even distribution over the 17 regions.

### Measuring novel antidiabetic use

We capture treatment choices for novel antidiabetic dual therapy as an outcome to determine whether patients have access to the conventional two-pill or the less complex single-pill treatment. We combined all products available in single- and two-pill treatments into one category irrespective to their active ingredient and brand.

We denote the physician’s prescription options to be the pair of outcomes for each patient $j$ of the study population. ${y}_{j}=0$ denotes that patient $j$ receives metformin in combination with one or more additional active ingredients as a two-pill treatment with one ingredient per pill (‘two-pill’). ${y}_{j}=1$ denotes the novel mixture of metformin and an additional ingredient in a single-pill (‘single-pill’).

The single-pill is available in the following combinations (Table 2): (1) Metformin and DPP-IV-inhibitors, (2) Metformin and sulfonylurea, or (3) Metformin and glitazones. The descriptive results of Table 2 indicate differences in the prescription prevalence of the single-pill. Single-pills account for about 35% of the oral antidiabetic prescriptions of private patients, compared to about 25% for public patients.

**Table 2:** Oral antidiabetic prescriptions for dual therapy by treatment pathway, stratified by insurance scheme and type of therapy

Therapy		Public Health Insurance		Private Health Insurance	
		*n*	%	*n*	%
Two-pill	Metformin +	366,610	39.57	16,517	30.91
	Sulfonylurea	191,093	20.62	6,118	11.45
	DPP-IV-Inhibitor	95,408	10.30	7,355	13.77
	Glinide	13,148	1.42	1,448	2.71
	GLP-1 Agonists	11,385	1.23	1,459	2.73
	Glitazones	2,874	0.31	848	1.59
	AGI	5,945	0.64	460	0.86
	SGLT2	4,007	0.43	506	0.95
	G/S	45	0.00	9	0.02
	*Total two-pill*	690,515	74.52	34,720	64.99
Single-pill	M-DPPIV	229,944	24.82	17,255	32.29
	M-Glitazones	4,615	0.50	1,432	2.68
	M-Sulfonylurea	1,444	0.16	24	0.04
	*Total single-pill*	236,003	25.48	18,711	35.01
	Observations	926,518		53,431	
	Patients	64,552		4,329	

Two-pill therapy indicates patients receiving metformin and an additional active ingredient in two different pills. Patients receiving only metformin are excluded from the sample

Data source: CEGEDIM MEDIMED prescription data 2011–2014

*Abbreviation: PHI* Private Health Insurance, *SHI* Statutory Health Insurance, *AGI* Alpha-glucosidase inhibitors, *SGLT2* inhibitor, Sodium-glucose cotransporter 2 inhibitor, *G/S* Glitazones/Sulfonylurea, *M-DPPIV* Single-pill containing metformin and DPP-IV-Inhibitor; M-Glitazones, Single-pill containing metformin and glitazones, M-Sulfonylurea, Single-pill containing metformin and sulfonylurea

### Empirical strategy

We aim to identify the effect of health insurance scheme on the probability of obtaining a single-pill compared to a two-pill treatment. We ideally would want to analyze if identical patients of different health insurance schemes would receive the same prescription medicine by the same physician. Since the prescription of a medicine happens in a face-to-face consultation, the risk of bias arises from the possibility that the physician remembers the patient. This bias makes a blinded, randomized experiment as is done in studies on inequalities in patient waiting times close to impossible [5, 52]. As a second best possible identification strategy, we account for any potential bias by considering confounders of the effect of health insurance scheme on the prescription of dual therapy treatments. We estimate fixed effect regression models by physician and region [53], clustering standard errors on physician level. Specifically, we estimate the following linear probability model (LPM):

1 $$P\left({{{S}}{{i}}{{n}}{{g}}{{l}}{{e}}-{{P}}{{i}}{{l}}{{l}}}_{{{i}}{{j}}}=1|{\alpha }_{i},{\delta }_{r}{,x}_{ij}\right)={\beta }_{1}{{{P}}{{H}}{{I}}}_{{{j}}}+{\beta }_{2}{Patient\ Age}_{j}+{\beta }_{3}{Patient\ Sex}_{j}+ {\beta }_{4 }Patient\ Risk\ {Score}_{j}+{\beta }_{5}{Quarter}_{ij}+ {\beta }_{6}PHI\ {Share}_{i}+{\alpha }_{i}+{\epsilon }_{ij}$$

where ‘Single-Pill’ denotes the binary outcome variable equaling to 1 if the single-pill is prescribed to a patient *j* by physician *i*. ‘PHI’ denotes the private health insurance scheme of patient *j* in comparison to public health insurance. ${\beta }_{1}$ reflects the probability to which the private health insurance scheme increases the probability of receiving the less complex treatment, ${x}_{ij}$ is a vector of control variables, including patient characteristics, unobserved physician heterogeneity, and timing of the treatment decision. ‘Patient Age’, ‘Patient Sex’ and ‘Patient Risk Score’ reflect patient related factors at level *j*. Diabetes prevalence is higher in men compared to women, and in older age groups [38]. The prescription-based ‘Patient Risk Score’ reflects the co-morbidity of the patient. ‘Quarter’ is a variable that controls for the quarter the patient *j* receives her first prescription by physician *i*. As we consider patients receiving access to antidiabetic treatments across time, we account for any time effects that might occur from early compared to late adopting physicians by including an indicator of the quarter the physician first prescribes the single-pill to the individual patient.

To assess the robustness of our estimates of ${\beta }_{1}$, we estimate separate variants of Eq. (1), most importantly to account for unobserved variation. Depending on the model, ${{\alpha }}_{{{i}}}$ is a fixed effect for physician $i$ to account for unobserved heterogeneity in practice style, ${{{\delta}}}_{{{r}}}$ is a fixed effect for region $r$ to account for unobserved heterogeneity of the distribution of patients with public or private insurance at regional level. We consider the region in which the physician practices to account for the unobserved differences in cost-control measures across the 17 physician associations, differences in the composition of the population by the health insurance schemes and diabetes prevalence. Moreover, there are considerable regional differences in diabetes prevalence in Germany, with higher rates (> 13%) in the counties of Eastern Germany compared to urban areas (for example in Hamburg 7.3%) [38].

In addition, we estimate Eq. (1) and account for unobserved heterogeneity in practice style of physician $i$ by including a physician-level fixed effect ${\alpha }$ [54]. Although we do not quantify treatment style by dimensions like aggressiveness or persistence, the physician-fixed effect controls for any bias from time-invariant variables of the unobserved preferences of the physician to prescribe single-pill compared to two-pill treatments [53, 55]. Finally, we estimate variants of the model that account for practice composition instead of physician fixed effects. The variable ‘PHI Share’ captures the absolute share of private patients in a practice *i* (Table 3, Model 4). ${\epsilon }_{ij}$ is the error term capturing unobserved environmental characteristics.

**Table 3:** Linear probability model of likelihood to receive single-pill treatment

	Model 1	Model 2	Model 3	Model 4	Model 5
*Private*	0.1267^***^	0.1074^***^	0.1033^***^	0.0973^***^	
*(ref: Public)*	(0.0104)	(0.0109)	(0.0072)	(0.0100)	
PHI Share					
< 5%				Ref	
5–10%				0.0270	
				(0.0219)	
10–15%				0.0658^**^	
				(0.0247)	
> 15%				0.0434	
				(0.0371)	
*Insurance scheme x* * Relative PHI share*					
Private Insurance, PHI share lower					0.1059^***^ (0.0136)
Private Insurance, PHI share higher					0.1241^***^ (0.0195)
Public Insurance, PHI share higher					0.0253 (0.0164)
Public Insurance, PHI share lower					Ref
Constant	0.5713^***^	1.0542^***^	1.3052^***^	1.0322^***^	1.0422^***^
	(0.0098)	(0.0480)	(0.0280)	(0.0514)	(0.0497)
Patient Characteristics	No	Yes	Yes	Yes	Yes
Quarter of first prescription	No	Yes	Yes	Yes	Yes
Region FE	No	Yes	No	Yes	Yes
Physician FE	No	No	Yes	No	No
N	68,355	68,355	68,355	68,355	68,355
R-squared	0.0038	0.0718	0.2060	0.0734	0.0724
F	147.1263	57.3184	38.6999	54.0553	55.4318
Prob > F	0.0000	0.0000	0.0000	0.0000	0.0000

Data source: CEGEDIM MEDIMED prescription data 2011–2014

*Abbreviations:* PHI, Private Health Insurance

* *p* < 0.05, ** *p* < 0.01, *** *p* < 0.001; standard errors in parentheses

To empirically test how practice composition compared to regional average affects the likelihood to receive the single-pill, we estimate separate regression models that examine the effect of the relative share of patients with private insurance in a practice on the effect of the patient’s insurance scheme, standard errors are clustered on physician level:

2 $$P\left({{{S}}{{i}}{{n}}{{g}}{{l}}{{e}}-{{P}}{{i}}{{l}}{{l}}}_{{{i}}{{j}}}=1|{\delta }_{r}{,x}_{ij}\right)={{{P}}{{H}}{{I}}}_{{{j}}}\ x\ {Relavtive\ PHI{ }{\ {S}}{{h}}{{a}}{{r}}{{e}}}_{i}+{\beta }_{1}{Patient\ Age}_{j}+{\beta }_{2}{Patient\ Sex}_{j}+ {\beta }_{3 }Patient\ Risk\ {Score}_{j}+{\beta }_{4}{Quarter}_{ij}+{{{\delta}}}_{{{r}}}+{\epsilon }_{ij}$$

with ${{{\delta}}}_{{{r}}}$ as a region fixed effect.Compared to Eq. (1), we include an interaction term of insurance scheme and patient composition in comparison with the regional average (‘Relative PHI Share’). ‘PHI’ captures the patient *j*’s insurance scheme. ‘Relative PHI Share’ is a binary variable that captures whether the difference of the share of private patients *j* in a practice *i* compared to the regional average of region $r$ is larger or smaller than zero. An interaction of both binary variables, results in coefficients for four possible scenarios:

- (1) a patient with private insurance in a practice with a low relative share of private patients
- (2) a patient with private insurance in a practice with a high relative share of private patients
- (3) a patient with public insurance in a practice with a high relative share of private patients
- (4) a patient with public insurance in a practice with a low relative share of private patients

To assess whether an effect might be driven by a specific combination of active ingredients, we estimate separate linear regression models by three groups of active ingredients that have a single-pill available. These are metformin plus DPP-IV-inhibitors, sulfonylurea, and glitazones. According to clinical guidelines, DPP-IV-inhibitors and sulfonylurea are equally suitable as a dual therapy if metformin monotherapy does not achieve the set therapeutic objective, yet both have disadvantages [44]. The combination of metformin and sulfonylurea has been associated with increased cardiovascular mortality, while DPP-IV-inhibitors have been associated with pancreatitis and pancreatic tumors [44, 56].

## Results

### Insurance scheme and treatment choice

We analyzed the channel of financial incentives to prescribe novel treatments and find that the insurance scheme of a patient has a significant effect on the likelihood to receive the less complex single-pill treatment (Table 3). The estimate of the LPM (Table 3, Model 1) reports the estimate for the baseline effect of private insurance, excluding all potential confounders. The estimated marginal effect is 0.1267 (*p* < 0.001). The low R-squared in Model 1 and Model 2 highlights the importance to account for physician specific characteristics. When accounting for patient characteristics like age, sex and comorbidity risk score, the timing at which the patient received the prescription and accounting for practice style variation in choosing oral antidiabetics across physicians (Model 3), the effect estimate of the probability to receive the novel single-pill treatment decreases to 0.1033 (*p* < 0.001), yet still positive and significant. This means, that patients with private insurance are about 10 percentage points more likely to receive the single-pill treatment compared to public patients. Adding physician level fixed effects is increasing the variance explained (Pseudo *R* ^2^ 0.206 compared to 0.0718 in Model 2). Yet not accounting for unobserved physician specific heterogeneity does not strongly bias our estimates of insurance scheme on the use of single-pill treatments. Accordingly, we cannot reject the hypothesis that patients with private insurance are more likely to obtain access to less complex treatments compared to patients with public insurance. In Model 3, we find a slight reduction in the standard errors compared to the other model specifications, as we compare the variation in the access to single-pill within one physician’s practice rather than between all patients.

### Practice composition and treatment choices

The second channel we analyzed was the influence of practice composition in dual practice. We find the practice composition to have significant influence on the likelihood to get the less complex treatment. When we account for the absolute share of private patients in the practice to account for the physician’s exposure to private patients (Model 4), the overall insurance effect slightly decreases to 0.0973 (*p* < 0.001). The estimates that account for absolute practice composition (Model 4) suggest that when the absolute share of private patients is around 10 to 15% the probability of receiving the single-pill treatment increases by 6.58 (*p* < 0.01) percentage points.

Table 4 shows the possible outcomes of a patient by public and private insurance scheme by practice composition relative to the regional average. We tested the hypothesis whether public patients in practices with relatively more private patients than the regional average had a higher likelihood to receive the single-pill treatment. The idea is that physicians provide “ready-to-wear” treatments that are suitable for the representative private patient. Public patients in practice settings with a low relative share of private patients represent the reference category. In line with Model 1–4 (Table 3), private patients have a higher probability of receiving the single-pill treatment in both practice settings, with either a low relative share (0.1059, *p* < 0.001) or a high relative share (0.1241, *p* < 0.001).

The effect of public patients visiting practices with a high relative share of patients with private health insurance share is 0.0253, indicating that public patients in a practice setting with a high relative share are more likely to receive the single-pill treatment. Yet this effect is not significant, thus, we cannot conclude that the treatment of public patients resembles the treatment of the privately insured such that more single-pill treatments are prescribed for the publicly insured.

Patients with private insurance are more likely to receive the novel single-pill treatment disparate of the practice composition. However, the differences in access between privately and publicly insured are larger in practices with a low relative share of privately insured (10.59 percentage points) compared to a high relative share (9.88 percentage points) (Table 4).

**Table 4:** Percentage point increase in receiving single-pill treatment—Interaction of a patient insurance schemes and practice composition

Insurance scheme Practice composition	Public	Private
Relative PHI share low	Ref (*n* = 63,353)	10.59^***^ (*n* = 2,541)
Relative PHI share high	2.53 (*n* = 39,820)	12.41^***^ (*n* = 4,814)

* *p* < 0.05, ** *p* < 0.01, *** *p* < 0.001 Results are based on Model 5 Table 3

*Abbreviation*: PHI, Private Health Insurance

The relative share is the difference between regional average share and the absolute proportion in a practice. A high relative share indicates that a practice has more private patients than the regional average, a low relative share vice versa

Data source: CEGEDIM MEDIMED prescription data 2011–2014

**Fig. 4:**
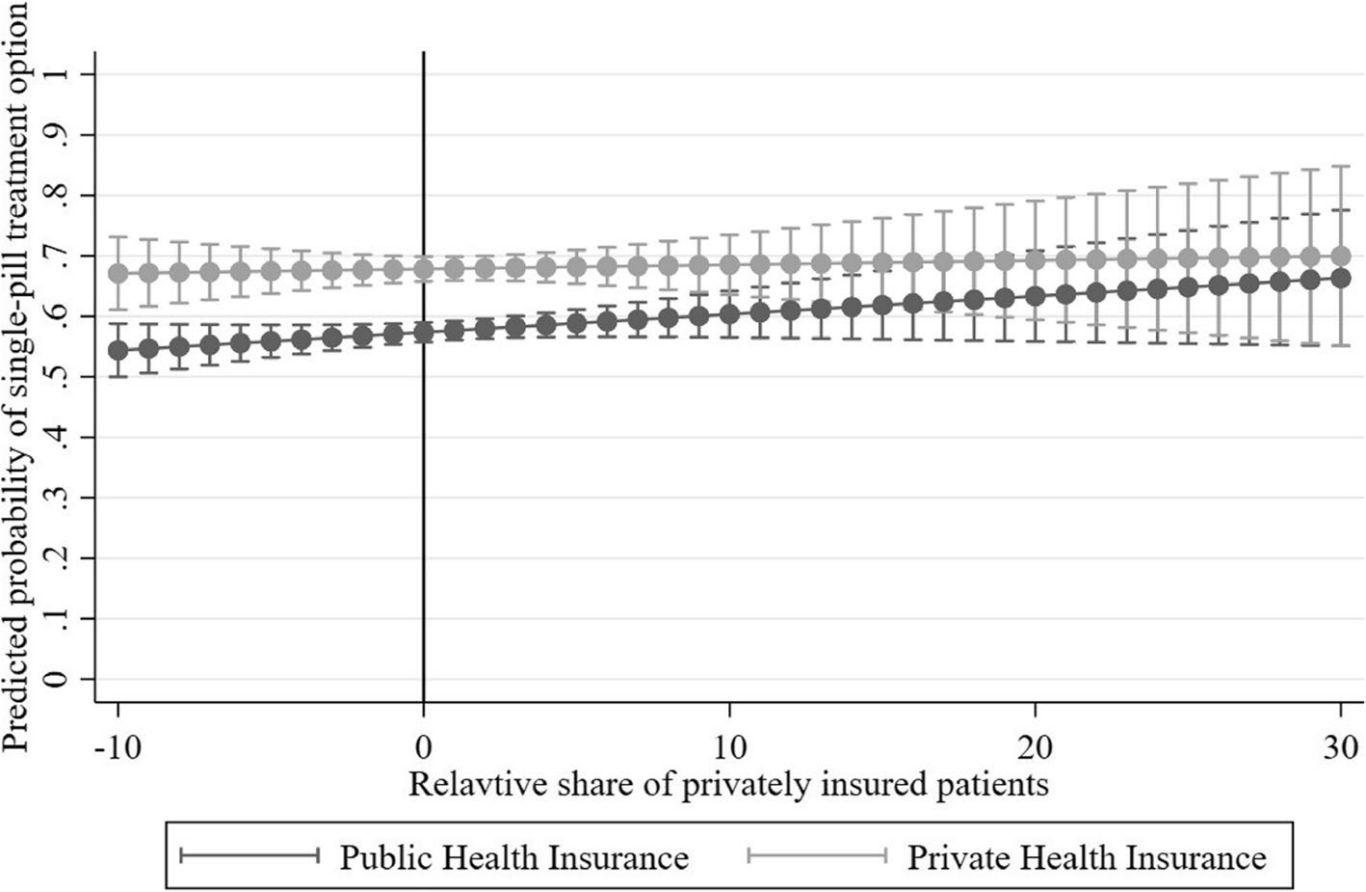
Linear probability to receive the single-pill treatment per health insurance scheme and relative share of private patients in a practice

Figure 4 presents the predicted probabilities to receive the single-pill treatment by the relative share of privately insured by insurance scheme. When the practice composition is equivalent to the regional average share of private patients, the probability for patients with public insurance to receive the single-pill treatment is 57.4% and 67.8% for private patients. The differences between public patients and patients with private insurance are largest (10.4 percentage points) in practice settings with a relative share around the regional average (at zero). We find that the probability of public patients to receive the single-pill is monotonically increasing by relative share of private patients in a practice. Especially, in settings where the relative share is very high (values above 20), the predicted probabilities to receive the single-pill treatment of private and public patients almost align, independent of the respective insurance scheme. On the contrary, if the relative share is very low, for example ‒10 percentage points, the difference in the probability to receive the single-pill increases to 54.4% for public patients compared to 67.1% for private patients. The probability of private patients remains constant around 67% independent of the relative share of private patients in a practice.

### Heterogeneity analyses

We assess whether the results are driven by the choice of the second ingredient besides metformin. The subgroup analysis for metformin plus DPP-IV-inhibitors and metformin plus sulfonylurea suggest homogeneous effects compared to our pooled estimates (see Tables A1 and A2 in the appendix). For the case that metformin and a DPP-IV-inhibitor is included in the single-pill, the effect of the health insurance scheme on the probability to receive the single-pill is 0.0157 (*p* < 0.05, Model 3, Table A1). When we consider metformin plus sulfonylurea, the effect estimate of the probability to receive the single-pill is 0.0644 (*p* < 0.001, Model 3, Table A1). This means that private patients independent of the second active ingredient in the single-pill are more likely to receive the single-pill.

When we test for the effect of the share of private patients in a practice (Model 5, Table A2), the insurance effect of private patients increases to a 9.06 percentage point (*p* < 0.001) higher probability of receiving the single-pill when they visit a practice that has a high relative share of private patients. We do not report effect estimates in the third group of metformin plus glitazones. *P*-values of the F-test indicated that none of the independent variables were statistically significant. This subgroup consisted of 2,596 patients and 477 physicians.

## Discussion

This study examined two channels of how different health insurance schemes impact treatment decisions in dual practice environments: firstly, the financial incentives promoting the prescription of innovative treatments, and secondly, the composition of practices in dual practice scenarios. The findings reveal that, within the same practice, individuals with private insurance enjoy enhanced access to less complex antidiabetic care compared to patients covered by public insurance. The estimated treatment effect of having private insurance indicates an about 10 percentage points higher probability to receive the single-pill treatment as oral antidiabetic therapy in Germany. This effect does not alter much when controlling for confounders including patient characteristics, unobserved regional or physician fixed-effects and the timing of the prescription decision.

We find the practice composition in dual practice settings to have significant influence on the likelihood to get the less complex treatment prescribed. The beneficial effect of the private insurance status on access to the single-pill is even larger in a practice with a high relative share of private patients (about 12 percentage points). The differences between publicly and private patients are largest in practices with a relative share lower or around the regional average of private patients, which is the most common practice setting in Germany. In this case, the probabilities for publicly and private patients to receive the single-pill differ by 10 percentage points.

While previous evidence comparing access to medical treatment per health insurance scheme has emphasized waiting times and utilization by the number of physician visits and hospitalizations [3, 5, 16], our results suggest that private patients have better access to novel treatments once they have entered the physician’s practice in a setting where private insurance imposes no cost-control on prescribing medicines. The average 10 percentage point difference that we identify poses an additional lever of access to receiving the best possible treatment even when a technology has reached substantial uptake levels [57]. The difference that we estimate is considerable as there are no direct financial incentives that would suggest this inequality to exist. As less complex therapy generally may be equally enhancing adherence to medication [58], our findings might explain why the privately insured utilize fewer physician visits [16].

Our results are in line with previous observations that private patients have better access to more novel therapy. Krobot et al. [59] assessed health insurance related barriers in accessing new migraine medicines in Germany developing a three-dimensional person-time-related hurdle model, albeit not controlling for the practice style of the physician and the regional distribution. In this study, patients with public insurance had a 2.4 times lower hazard to receive initial migraine therapy compared to their privately insured counterparts. Additionally, we expand the descriptive evidence that patients with private insurance in Germany proportionally receive more innovative as well as more expensive medicines, in particular, for medicines with proven added value [60].

Although the oral antidiabetic treatments that we study are very similar in terms of their effectiveness to control T2D, the single-pill has a potential to improve the adherence behavior of patients, as it entails a less complex regimen. Evidence from retrospective cohort studies investigating secondary adherence suggests that a single-pill shows higher adherence, in particular, in patients who receive dual therapy. This applies to both, patients switching from monotherapy to dual-therapy and to patients switching from a two-pill treatment to a single-pill [8, 10]. Literature finds, that more complex medication regimen reduce medication adherence [61] and, in particular for patients with T2D, a high medication count and a complex medication complexity is associated with poor glycemic control, with medication adherence being the mediator between the two [62]. Studies have assessed consequences of non-adherence in different chronic conditions. It was found that T2D patients that are non-adherent are more than twice as likely to be hospitalized [63] and that despite the increase of prescription costs through better adherence, the overall healthcare costs decrease as hospitalization and emergency department usage goes down [64]. Even though we do not have ex ante adherence rates of the patients, adherence is multidimensional such that a patient’s insurance status alone would not be sufficient to extract differences [65]. However, we cannot assess long-term health outcomes like mortality due to differential treatments, as we are using data from ambulatory care not covering the decease of a patient.

The findings of this study are still applicable in 2023, as the German health insurance system did not undergo substantial changes since the ending of our observation period, that means 2014. Metformin is still the first line choice in T2D treatment along with a number of combination of therapies [66]. Moreover, our empirical approach can be adopted to new treatment options like semaglutide or exenatide that have received raised attention due to the potential long-term weight loss benefits and ease in adhering to anti-diabetic therapy through once-weekly injections. The progressive nature of T2D therapy often involves step-up regimen through combination of different active ingredients [67, 68]. More complex treatments demand even closer attention in ensuring equal access, highlighting the applicability of this study’s research aim.

We find a small effect of the effect of the patient’s health insurance scheme on receiving metformin plus a DPP-IV-inhibitor as a single-pill. This might be explained by the lack of added benefit of some medicines with DPP-IV-inhibitors, according to prescription guidelines for DPP-IV-inhibitors by the Federal Joint Committee [56]. Clinical guidelines state DPP-IV-inhibitors and sulfonylurea as interchangeable, yet both have stated clinical limitations [44]. The combination of metformin and sulfonylurea has been associated with increased cardiovascular mortality, while DPP-IV-inhibitors have been associated with pancreatitis and pancreatic tumors [44, 56]. Sulfonylurea is usually the second ingredient when metformin monotherapy is insufficient, yet this is not in line with clinical guidelines [69].

Publicly insured patients do not have advantageous access to the single-pill in settings with a practice composition of a high relative share of private patients compared to practices with a low share. This shows, that physicians customize their treatments or choose treatments for the mean patient within one insurance category. This finding is in line with Glied and Zivin [32], suggesting that physicians vary fixed and variable costs by practice composition. The fixed costs of physicians include investments for equipment or practice capacity. Fixed components do not vary across patients with different insurance scheme and, are set by the physician in advance. Such choices can be based on practice composition, as physicians with a high share of private patients will likely make other investment decisions. Variable costs of physician’s may include waiting times till appointment, time spent with the patient, and the scope of services provided or medicines prescribed. In a practice setting with a high share of privately insured, publicly insured are likely to receive lower variable costs to compensate fixed costs set for a practice composition with a high share of patients with private insurance share. Vice versa, privately insured in practice settings with relatively few privately insured and lower fixed costs receive more variable effort to compensate [32]. For the United States, Glied and Zivin [32] find that practices with a higher share of managed care patients (e.g. publicly insured) treated fee-for-service (e.g. privately insured) and managed care patients about similar. In contrast to this, when health insurance schemes differ by management and monitoring of prescription behavior, the private patients are more likely to receive the single-pill disparate of the practice composition. This shows, that despite the practice composition, physicians are optimizing variable costs as well.

The data used in this study limits us to completely rule out if patients with variable socioeconomic backgrounds beyond age, gender and comorbidity demand the single-pill. Cutler et al. [2] finds the influence of patients on physician treatment variation to be small. One can argue that this is the case in the German setting as well. The drug advertising law (“Heilmittelwerbegesetz”) prohibits the advertisement of prescription drugs to patients, thus it is unlikely that a layperson knows of pharmaceutical alternatives to their treatment and specifically demands such alternative [70]. The existing information asymmetry between physician and patient demands that the physician uses her information advantage to provide the most suitable and beneficial treatment in the best of the patient’s needs [71, 72]. Hence, if our measured supply-effect is biased by patient demand, it would mean that physicians are not moderating patient demand adequately.

One threat to our empirical identification strategy is potential selection by patients opting into treatment to receive the single-pill instead of the two-pill treatment. This means that patients would change their insurance scheme from public to private insurance to receive the less complex treatment. Considering the possibilities to switch health insurance scheme, this, however, is an unlikely event in our context. Patients face a compulsory income threshold to opt into private insurance, including age and health assessment to define the individual monthly premium [16]. This leads to patients switching, if at all, at a healthier stage. Second, as T2D is a chronic condition, it is unlikely that patients opt into private insurance once diagnosed, as it would come along with much higher monthly premiums. Therefore, we consider this potential bias to be small. In our sample, there are no T2D patients switching insurance supporting that patient incentives to switch insurance are low once receiving antidiabetic prescriptions.

Another limitation is that, although, oral antidiabetics are equally reimbursed in private and public insurance, prices and corresponding rebates might influence physician choices. The data we analyze allows observing gross prices excluding rebates only. Physicians are price sensitive in their prescription behavior and account for costs when information on medicine prices is transparent [73]. In the German context, to write prescriptions, manage medicine budgets and compliance with cost-control measures, physicians observe gross prices and cannot infer the negotiated rebates of the public insurance schemes. Additionally, we cannot observe when physicians choose the two-pill treatment to flexibly adjust dosages of ingredients. Clinical guidelines suggest that dual therapy is advised if metformin monotherapy is not achieving the set treatment goals in Germany [44].

## Conclusion

This study analyzed the effects of health insurance schemes on medical treatment decisions that determine access to novel therapeutic pathways in T2D patients. We find unequal access to antidiabetic care for patients based on insurance schemes within the same physician practice. Patients that hold private insurance are about 10 percentage points more likely to receive a treatment with a less complex medication regimen compared to patients with public insurance. The inequality in access that we document is largest in settings with a practice composition with a share of private patients around the regional average, thus in most German ambulatory practices. Our results show that even when there is a predominant health insurance scheme (i.e. public health insurance) that covers 90% of the population, there is unequal access to novel therapies. Our findings contribute to the literature that considers treatment inequality due to indirect financial incentives that shape physician treatment behavior.

### Supplementary Information

**Additional file 1: Table A1.** Linear probability model of likelihood to receive the single-pill treatment - DPP-4 Inhibitors. **Table A2.** Linear probability model of likelihood to receive the single-pill treatment – Sulfonylurea.

## Data Availability

The data used in this study include patient-level prescription data that are covered by a non-disclosure agreement. Data on physicians’ decisions to prescribe a novel treatment were taken from the CEGEDIM MEDIMED prescriber panel. Information on data access options can be found at https://www.medimed.info/. Last access of the project team was on April 29th, 2021. Data to classify the prescribed product by active ingredient were taken from the 2016 EphMRA/PBIRG Anatomical Classification. This can be downloaded from the European Pharmaceutical Market Research Association as a pdf file and information was extracted in machine readable format by the authors. Available at: https://www.ephmra.org/media/1090/atcguidelines2016final.pdf

## Abbreviations

T2D: Type 2 diabetes
LPM: Linear probability model
PHI: Private health insurance
GLP-1-agonists: Glucagon-like peptide-1 receptor agonists
SGLT2-inhibitors: Sodium-glucose cotransporter 2 inhibitors
DPP-IV-inhibitors: Dipeptidyl peptidase IV inhibitors
HBA1c: Blood glycated hemoglobin levels

## Footnotes

1 In comparison to Pratt et al. [51], the comorbidity risk score used in this study has 45 comorbidity classes in total as some ATC codes indicate different comorbidity classes by disease category based on diagnosis data. As our data set does not include diagnosis codes, the patient risk score varies between zero and 45 instead of 0 and 46.
