# Supplementary material for: Insurance barriers and inequalities in health care access: evidence from dual practice
Source: Health Econ Rev. 2024 Mar 21;14:23. doi: 10.1186/s13561-024-00500-y (PMC10956272; doi:10.1186/s13561-024-00500-y)
Supplement: Supplementary file 1 — Additional file 1: Table A1. Linear probability model of likelihood to receive the single-pill treatment - DPP-4 Inhibitors. Table A2. Linear probability model of likelihood to receive the single-pill treatment – Sulfonylurea. [file 13561_2024_500_MOESM1_ESM.docx]

**Supplementary Material**

**Appendix**

Table A1 - Linear probability model of likelihood to receive the single-pill treatment - DPP-4 Inhibitors

|  | Model 1 | Model 2 | Model 3 | Model 4 | Model 5 |
| --- | --- | --- | --- | --- | --- |
|  |  |  |  |  |  |
| *Private* | 0.0141 | 0.0072 | 0.0157^*^ | 0.0049 |  |
| *(ref: Public)* | (0.0088) | (0.0087) | (0.0071) | (0.0082) |  |
|  |  |  |  |  |  |
| PHI Share |  |  |  |  |  |
| <5% |  |  |  | Ref. |  |
|  |  |  |  |  |  |
| 5-10% |  |  |  | -0.0060 |  |
|  |  |  |  | (0.0161) |  |
| 10-15% |  |  |  | 0.0240 |  |
|  |  |  |  | (0.0176) |  |
| >15% |  |  |  | 0.0078 |  |
|  |  |  |  | (0.0270) |  |
| *Insurance scheme x*  *Relative PHI share* | |  |  |  |  |
|  |  |  |  |  |  |
| Private Insurance,  PHI share lower |  |  |  |  | 0.0168  (0.0122) |
|  |  |  |  |  |  |
| Private Insurance,  PHI share higher |  |  |  |  | 0.0048  (0.0161) |
|  |  |  |  |  |  |
| Public Insurance,  PHI share higher |  |  |  |  | 0.0051  (0.0119) |
|  |  |  |  |  |  |
| Public Insurance,  PHI share lower |  |  |  |  | Ref. |
|  |  |  |  |  |  |
| Constant | 0.7614^***^ | 1.0127^***^ | 1.1388^***^ | 1.0110^***^ | 1.0102^***^ |
|  | (0.0065) | (0.0326) | (0.0254) | (0.0346) | (0.0335) |
|  |  |  |  |  |  |
| Patient Characteristics | No | Yes | Yes | Yes | Yes |
| Quarter of first prescription | No | Yes | Yes | Yes | Yes |
| Region FE | No | Yes | No | Yes | Yes |
| Physician FE | No | No | Yes | No | No |
| N | 50,906 | 50,906 | 50,906 | 50,906 | 50,906 |
| R-squared | 0.0001 | 0.0316 | 0.1319 | 0.0320 | 0.0317 |
| F | 2.5883 | 20.7614 | 13.2146 | 19.0228 | 19.8215 |
| Prob > F | 0.0000 | 0.0000 | 0.0000 | 0.0000 | 0.0000 |

Note: * p<0.05, ** p<0.01, *** p<0.001; standard errors in parentheses

Abbreviations: PHI, Private Health Insurance

Data source: CEGEDIM MEDIMED prescription data 2011-2014

Table A2 - Linear probability model of likelihood to receive the single-pill treatment – Sulfonylurea

|  | Model 1 | Model 2 | Model 3 | Model 4 | Model 5 |
| --- | --- | --- | --- | --- | --- |
|  |  |  |  |  |  |
| *Private* | 0.0647^***^ | 0.0761^***^ | 0.0644^***^ | 0.0684^***^ |  |
| *(ref: Public)* | (0.0187) | (0.0179) | (0.0159) | (0.0171) |  |
|  |  |  |  |  |  |
| PHI Share |  |  |  |  |  |
| <5% |  |  |  | Ref. |  |
|  |  |  |  |  |  |
| 5-10% |  |  |  | 0.0046 |  |
|  |  |  |  | (0.0146) |  |
| 10-15% |  |  |  | 0.0596^**^ |  |
|  |  |  |  | (0.0218) |  |
| >15% |  |  |  | 0.0261 |  |
|  |  |  |  | (0.0303) |  |
| *Insurance scheme x*  *Relative PHI share* | |  |  |  |  |
|  |  |  |  |  |  |
| Private Insurance,  PHI share lower |  |  |  |  | 0.0640^*^  (0.0273) |
|  |  |  |  |  |  |
| Private Insurance,  PHI share higher |  |  |  |  | 0.0906^***^  (0.0260) |
|  |  |  |  |  |  |
| Public Insurance,  PHI share higher |  |  |  |  | 0.0116  (0.0128) |
|  |  |  |  |  |  |
| Public Insurance,  PHI share lower |  |  |  |  | Ref. |
|  |  |  |  |  |  |
| Constant | 0.2275^***^ | 0.6982^***^ | 0.9358^***^ | 0.6912^***^ | 0.6949^***^ |
|  | (0.0068) | (0.0414) | (0.0750) | (0.0404) | (0.0411) |
|  |  |  |  |  |  |
| Patient Characteristics | No | Yes | Yes | Yes | Yes |
| Quarter of first prescription | No | Yes | Yes | Yes | Yes |
| Region FE | No | Yes | No | Yes | Yes |
| Physician FE | No | No | Yes | No | No |
| N | 25,578 | 25,578 | 25,578 | 25,578 | 25,578 |
| R-squared | 0.0007 | 0.0542 | 0.1635 | 0.0556 | 0.0544 |
| F | 11.9562 | 26.5234 | . | 24.6627 | 25.6897 |
| Prob > F | 0.000 | 0.0000 | 0.0000 | 0.0000 | 0.0000 |

Note: * p<0.05, ** p<0.01, *** p<0.001; standard errors in parentheses

Abbreviations: PHI, Private Health Insurance

Data source: CEGEDIM MEDIMED prescription data 2011-2014
